# The *TaGSK1*, *TaSRG*, *TaPTF1,* and *TaP5CS* Gene Transcripts Confirm Salinity Tolerance by Increasing Proline Production in Wheat (*Triticum aestivum* L.)

**DOI:** 10.3390/plants11233401

**Published:** 2022-12-06

**Authors:** Murat Aycan, Marouane Baslam, Toshiaki Mitsui, Mustafa Yildiz

**Affiliations:** 1 Graduate School of Natural and Applied Sciences, Ankara University, Ankara 06110, Türkiye; 2 Laboratory of Biochemistry, Faculty of Agriculture, Niigata University, Niigata 950-2181, Japan; 3 Department of Field Crops, Faculty of Agriculture, Ankara University, Ankara 06110, Türkiye

**Keywords:** salt stress, proline-related genes, gene expression, qRT-PCR, correlation network analysis

## Abstract

Salinity is an abiotic stress factor that reduces yield and threatens food security in the world’s arid and semi-arid regions. The development of salt-tolerant genotypes is critical for mitigating yield losses, and this journey begins with the identification of sensitive and tolerant plants. Numerous physiologic and molecular markers for detecting salt-tolerant wheat genotypes have been developed. One of them is proline, which has been used for a long time but has received little information about proline-related genes in wheat genotypes. In this study, proline content and the expression levels of proline-related genes (*TaPTF1*, *TaDHN*, *TaSRG*, *TaSC*, *TaPIMP1*, *TaMIP*, *TaHKT1;4*, *TaGSK*, *TaP5CS*, and *TaMYB*) were examined in sensitive, moderate, and tolerant genotypes under salt stress (0, 50, 150, and 250 mM NaCl) for 0, 12, and 24 h. Our results show that salt stress increased the proline content in all genotypes, but it was found higher in salt-tolerant genotypes than in moderate and sensitive genotypes. The salinity stress increased gene expression levels in salt-tolerant and moderate genotypes. While salt-stress exposure for 12 and 24 h had a substantial effect on gene expression in wheat, *TaPTF1*, *TaPIMP1*, *TaMIP*, *TaHKT1;4*, and *TaMYB* genes were considerably upregulated in 24 h. The salt-tolerant genotypes showed a higher positive interaction than a negative interaction. The *TaPTF1*, *TaP5CS*, *TaGSK1*, and *TaSRG* genes were found to be more selective than the other analyzed genes under salt-stress conditions. Despite each gene’s specific function, increasing proline biosynthesis functioned as a common mechanism for separating salt tolerance from sensitivity.

## 1. Introduction

Salinity is a major abiotic stressor that has a negative impact on seed germination, plant growth, and productivity in arid and semi-arid regions [1]. Saline soils are defined as having electrical conductivity (EC) of the saturation soil-paste extract greater than 4 dS/m at 25 °C, which corresponds to approximately 40 mM NaCl and generates an osmotic pressure of approximately 0.2 MPa [2]. It is estimated that salt stress affects approximately 800 million hectares or 6.5% of the world’s total land area, and salt has already damaged 45 million ha (19.5%) of the current 230 million ha of irrigated land [3]. High salinity problems are expected to affect approximately 50% of total arable agricultural land by 2050 [4]. Soil salinity is significant abiotic stress that has a devastating impact on crop production [5]. Salinity-related yield reduction in crops threatens food security with rapid growth in the world’s population, which is estimated to reach 9 billion by the end of the next 30 years [6]. Currently, projections show that approximately 690 million (11% of the global population) are hungry, and food demand is expected to increase by 85% (approximately 2.7 billion people) by 2050 [7,8].

Wheat plays a critical role in ensuring food and nutritional security; however, rapidly rising soil and water salinity pose a serious threat to global wheat production [9]. Wheat, like other glycophytes, is severely impacted by high salinity levels in soils, with 28–60% losses in grain yield under saline conditions [10,11]. Improving salt tolerance is one of the most effective and feasible strategies for reducing the negative effects of salinity on wheat production [12]. Several morphological, physiological, and molecular markers have been developed to assort salt-tolerant genotypes. The assessment of genetic variability using morphological and physiological markers is useful for early field-based characterization and selection, but these markers are heavily influenced by environmental fluxes [13]. Studies need to be supplemented with more robust genetically linked molecular and biochemical markers for selection precision in order to validate the morphological and physiological marker-based data [14]. Many tolerant genotypes have now been determined thanks to advances in molecular markers and modern genomic techniques [15].

Many studies have been conducted to investigate salt-tolerant genotypes in Arabidopsis and rice and their application in breeding programs, with the goal of identifying salt-tolerance genes [16]. Although most salinity-tolerance-related genes in Arabidopsis and rice have been identified, not enough genes have been identified in wheat due to the genome size and an insufficient gene map [17]. The genes identified in wheat were also investigated in *Arabidopsis* plants by transgenic studies [18,19,20,21]. Proline is one of the most significant amino acids synthesized and stored in the plant in response to salt stress, and proline accretion is a sign of abiotic stress tolerance in plants using different stress signaling pathways (phytohormones, calcium signaling, and mitogen-activated protein (MAP) kinase pathways) [22,23,24]. Proline has been shown to promote plant growth, physiology, biochemistry, anatomical features, and antioxidant system defense when exposed to salinity stress [25]. Proline is made from glutamate in the glutamate pathway by enzymes called 1-pyrroline-5-carboxylate synthetases (P5CS) and Δ1-pyrroline-5-carboxylate reductase (P5CR) genes [26,27]. Although the *TaP5CS* gene is a crucial gene for proline production, many other main genes such as pyrroline-5-carboxylate (P5C), pyrroline-5-carboxylate reductase (P5CR), and pyrroline-5-carboxylate dehydrogenase (P5CDH) have been found to also contribute to the ability to produce proline in plants [24]. Proline accumulation-related genes were previously identified utilizing the gene expression approach, which is widely utilized in this field [28,29,30,31,32]. Among identified genes, transcription factors (TFs) are involved in a variety of biological processes, including the regulation of stress tolerance [33]. MYB proteins are one of the most important TFs families in plants, and it is a key regulator gene involved in salt-stress adaptation in wheat [34,35]. ABA regulates the *TaMYB* and putative integral membrane protein 1 (*TaPIMP1*) genes, and *TaPIMP1* overexpression boosted proline synthesis, resulting in increased drought tolerance [36]. The pi starvation-induced TF 1 (*TaPTF1*) gene is also a member of the basic helix–loop–helix TFs (bHLH) family, which improves plant salinity tolerance by increasing proline production [37]. In response to salt stress, the high-affinity K^+^ transporter (*TaHKT1;4*) gene aids in the exclusion of Na^+^ from leaf blades [38], and overexpression of the glycogen synthase kinase (*TaGSK1*) and salt response gene (*TaSRG*) increases salt tolerance by the reduction in the amount of Na^+^ in cells and proline accumulation [20,39]. An aquaporin gene NOD26-like membrane integral protein (*TaMIP*) accumulates higher K^+^ and proline content and lower Na^+^ concentration in salt-exposed plants when *TaMIP* is overexpressed [19]. The dehydrin (*TaDHN*) and salt-tolerant correlate (*TaSC*) genes, which regulate the rate-limiting stage of glutamate-derived proline biosynthesis, rapidly increase in mRNA levels in reaction to salt [21,40].

In this study, we aimed to test proline content and the activity of *TaPTF1*, *TaDHN*, *TaSRG*, *TaSC*, *TaPIMP1*, *TaMIP*, *TaHKT1;4*, *TaGSK1*, *TaP5CS*, and *TaMYB* genes in two salt-sensitive, two moderate, and two salt-tolerant wheat genotypes under 0, 50, 150, and 250 mM NaCl stress conditions for 0, 12, and 24 h.

## 2. Results

The three-way analysis of variance (ANOVA) test was used to distinguish differences in proline content and the gene expression of *TaPTF1*, *TaDHN*, *TaSRG*, *TaSC*, *TaPIMP1*, *TaMIP*, *TaHKT1;4*, *TaGSK*, *TaP5CS*, and *TaMYB* genes with varying Time (T; 0, 12, and 24 h), Salt levels (S; 0, 50, 150, and 250 mM), Genotypes (G; two salt-sensitive, moderate, and salt-tolerant), and their interactions (T × S, T × G, S × G, and T × S × G) (Table 1 and Table S2). The three-way ANOVA results revealed that T × S, T × G, S × G, and T × S × G interactions significantly (*p* < 0.001) affected proline content and all measured gene expressions.

### 2.1. Proline Content and Phylogenetic Tree of Genes

**Figure 1:**
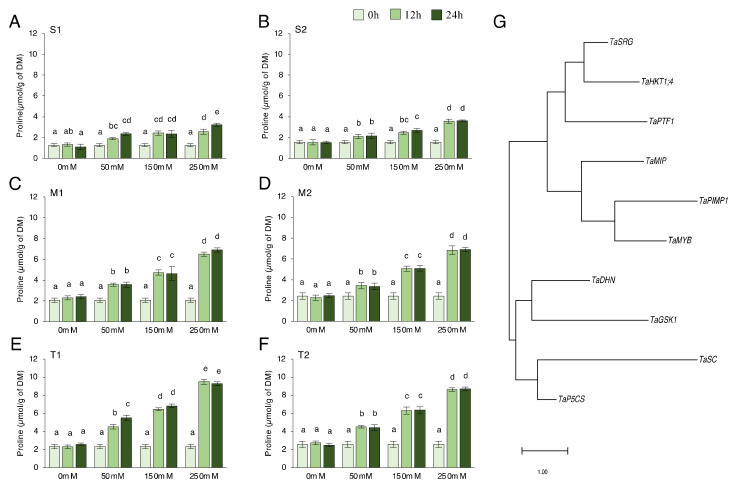
Proline content and phylogenetic tree of genes. (**A**) The proline content of the salt-sensitive-1 (S1), (**B**) sensitive-2 (S2), (**C**) moderate-1 (M1), (**D**) moderate-2 (M2), (**E**) tolerant-1 (T1), (**F**) tolerant-2 (T2), and (**G**) evolutionary phylogenetic tree of *TaPTF1*, *TaDHN*, *TaSRG*, *TaSC*, *TaPIMP1*, *TaMIP*, *TaHKT1;4*, *TaGSK*, *TaP5CS*, and *TaMYB* genes by using amino acid sequences in MEGA 11.0.13 software. The proline content in S1, S2, M1, M2, T1, and T2 genotypes exposed to 0, 50, 150, and 250 mM NaCl stress for 0, 12, and 24 h. Means (±standard deviation) within the same graph followed by letters are significantly different at *p* < 0.05 according to the Tukey HSD test from independent biological triplicates (*n* = 3).

**Figure A1:**
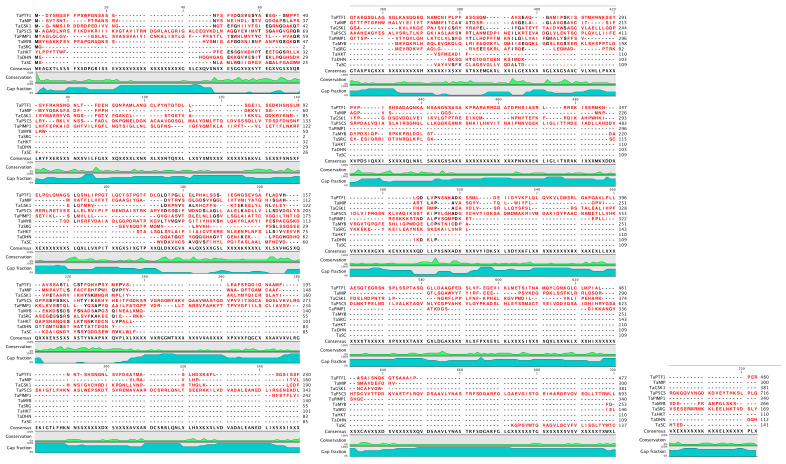
Amino acid alignment of *TaPTF1*, *TaDHN*, *TaSRG*, *TaSC*, *TaPIMP1*, *TaMIP*, *TaHKT1;4*, *TaGSK*, *TaP5CS*, and *TaMYB* genes.

We checked proline content in salt-sensitive, moderate, and salt-tolerant genotypes under 0, 50, 150, and 250 mM NaCl for 0, 12, and 24 h. Proline content significantly increased under salt-stress conditions. The proline production after 12 and 24 h in almost all genotypes was found to not be statistically different. The moderate and tolerant genotypes generated more proline than the sensitive genotypes, but the proline concentration of the tolerant genotype was found to be higher (Figure 1A–F). In the S1 genotype, the proline content increased by a minimum of 50% after 12 h of 50 mM NaCl stress and a maximum of 155% after 24 h of 250 mM NaCl stress (Figure 1A). It increased by a minimum of 34% after 12 h of 50 mM NaCl stress and a maximum of 129% after 24 h of 250 mM NaCl stress in the S2 genotype (Figure 1B). In the M1 genotype, proline content increased by a minimum of 72% after 12 h of 50 mM NaCl stress and a maximum of 233% after 24 h of 250 mM NaCl stress (Figure 1C). It increased by a minimum of 41% after 12 h of 50 mM NaCl stress and a maximum of 183% after 24 h of 250 mM NaCl stress in the M2 genotype (Figure 1D). The proline content increased by a minimum of 94 % at 12 h of 50 mM NaCl stress and a maximum of 298 % at 24 h of 250 mM NaCl stress in the T1 genotype (Figure 1E). It was increased by a minimum of 76 % at 12 h of 50 mM NaCl stress and a maximum of 238 % at 24 h of 250 mM NaCl stress in the T2 genotype (Figure 1F). The higher proline content was measured in the T1 genotype under 250 mM NaCl stress conditions (Figure 1E). The proline content of salt-sensitive genotypes was almost doubled, and a triple increment was seen in the moderate and tolerant genotypes. To explore the phylogenetic relationship among proline-related genes in *Triticum aestivum* L., a rooted maximum-likelihood phylogenetic tree with 10 (*TaPTF1*, *TaDHN*, *TaSRG*, *TaSC*, *TaPIMP1*, *TaMIP*, *TaHKT1;4*, *TaGSK*, *TaP5CS*, and *TaMYB*) genes (Figure 1G) was inferred from the amino acid sequences (Table S2, Figure A1) using the MEGA 11.0.13 program [41]. The genes can be subdivided into two main clusters and four well-conserved subclusters with the highest log likelihood (−2352.72). The evolutionary phylogenetic tree showed that *TaP5CS* and *TaSC*, *TaGSK1,* and *TaDHN* genes were found in the same cluster. *TaPIMP1* and *TaMYB*, *TaSRG,* and *TaHKT1;4* genes were found close to each other (Figure 1G).

### 2.2. Expression Profiles of Proline-Related Genes

After the detection of the proline accumulation profile, we examined changes in the gene expression profile of proline-related genes (*TaPTF1*, *TaDHN*, *TaSRG*, *TaSC*, *TaPIMP1*, *TaMIP*, *TaHKT1;4*, *TaGSK*, *TaP5CS*, and *TaMYB*) in all tested sensitive, moderate, and tolerant plants.

#### 2.2.1. *TaPTF1* and *TaDHN* Genes

**Figure 2:**
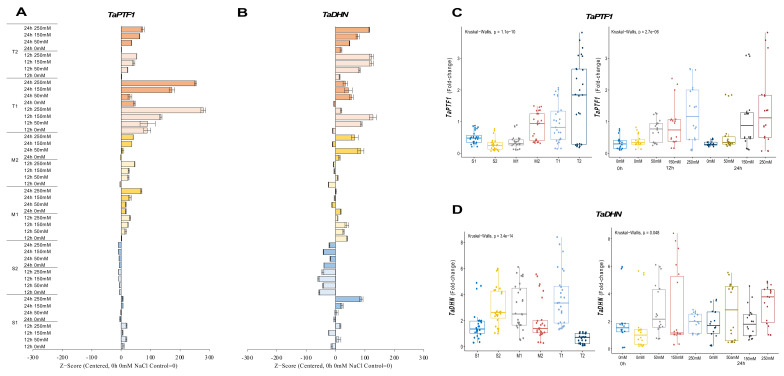
The expression of *TaPTF1* and *TaDHN* genes. (**A**) The gene expression pattern of *TaPTF1* and (**B**) *TaDHN* genes; (**C**) the total gene expression profile of *TaPTF1* for genotypes, salt stress, and time course; (**D**) the total gene expression profile of *TaDHN* for genotypes, salt stress, and time course. The Cq values formed the basis of qRT-PCR. The reference gene *TaACTIN* was used to standardize the Cq value for each sample. Z-score centered to 0 h 0 mM treatment (Control = 0). Values represent the means ± SD (*n* = 3).

**Figure A2:**
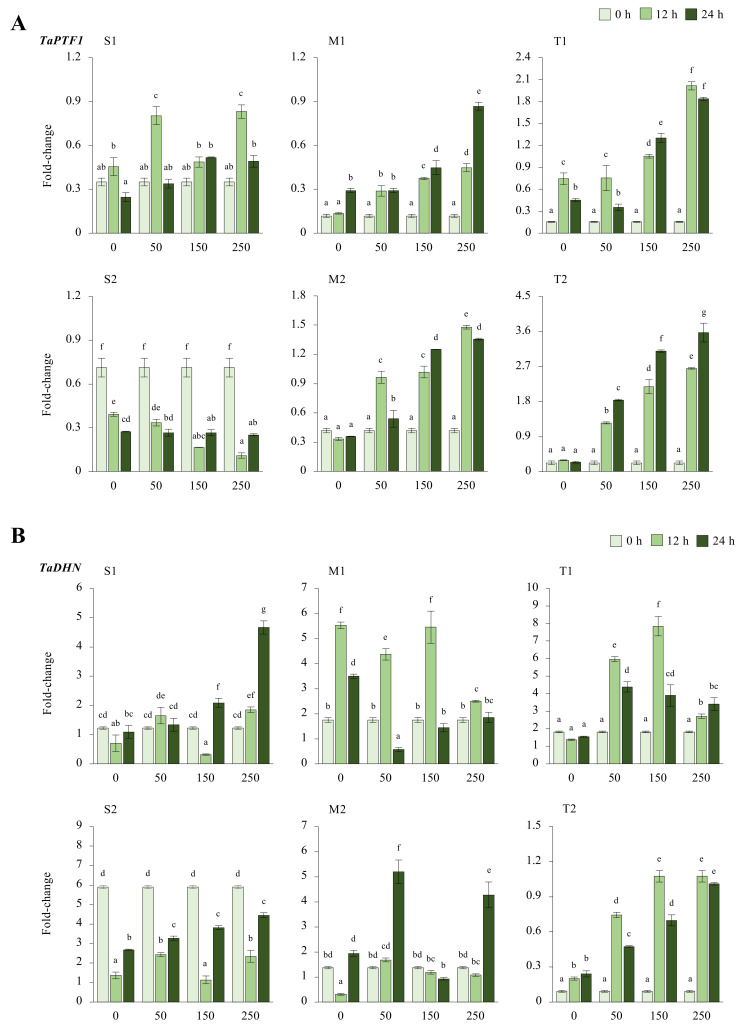
(**A**) The gene expression of the *TaPTF1* and (**B**) *TaDHN* in the leaves of salt-sensitive (S1), S2, moderate (M1), M2, -tolerant (T1), and T2 genotypes exposed to 0, 50, 150, and 250 mM NaCl stress for 0, 12, 14 h. The Cq values formed the basis of qRT-PCR. The reference gene *TaACTIN* was used to standardize the Cq value for each sample. Means (±standard deviation) within the same graph followed by letters are significantly different at *p* < 0.05 according to the Tukey HSD test from independent biological triplicates (*n* = 3).

The expression of *TaPTF1* was decreased from 0.7-fold (0 h of 0 mM NaCl—control) up to 0.1-fold (12 h of 250 mM NaCl) in the salt-sensitive-2 (S2) genotype under salt-stress conditions. Increasing salt doses (0 to 250 mM NaCl) also negatively affected mRNA transcript levels in the S2 genotype, but the transcript level of the S1 genotype slightly increased from 0.3-fold at control (0 h of 0 mM NaCl) to 0.8-fold at 12 h of salt stress under the applications of 50 and 250 mM NaCl. The moderate (M1 and M2) and tolerant (T1 and T2) genotypes showed higher increments in transcript level under salt-stress conditions. The M1 genotypes increased mRNA transcript levels from 0.1-fold at control to 0.8-fold at 24 h of 250 mM NaCl stress and from 0.4-fold at control to 1.4-fold at 12 h of 250 mM NaCl stress in the M2 genotype. The *TaPTF1* transcript increased from 0.1-fold (control) to 2-fold at 12 h of 250 mM NaCl stress in the M1 genotype and from 0.2-fold (control) to 3.5-fold under 250 mM for 24 h in M2 genotype (Figure 2A and Figure A2A). The T2 genotype had a higher total fold-change in *TaPTF1* gene expression. The *TaPTF1* gene transcript fold-change was found to be higher under stress conditions of 12 h of 150 and 250 mM NaCl stresses (Figure 2C).

The transcript level of the *TaDHN* gene was increased from 0.1-fold (control) to 4.6-fold and 1.8-fold at 24 h of 150 and 250 mM NaCl in the S1 genotype, respectively. However, the mRNA transcripts were decreased in the S2 genotype from 5.8-fold at control to 7.4 and 1.9-fold under 150 mM NaCl stress for 12 and 24 h, respectively. The M1 genotype showed a significant increment from 1.7-fold (control) to 5.4-fold at 12 h of 150 mM salt stress, and *TaDHN* expression was increased from 1.3-fold (control) to 5.1- and 4.2-fold in M2 genotypes under 50 and 250 mM NaCl for 24 h. T1 genotypes showed a significant increment in *TaDHN* expression from 1.8-fold at control to 5.9- and 7.8-fold at 12 h of 50 and 150 mM NaCl stress conditions, respectively. Similarly, *TaDHN* gene transcripts were increased from 0.09-fold (control) to 1.7-fold at 12 h of 150 and 250 mM NaCl stress in the T2 genotype (Figure 2B and Figure A2B). The T1 genotype had a higher total expression fold-change, and 12 h of 150 mM NaCl had the highest expression fold-change compared to all other treatments (Figure 2D).

#### 2.2.2. *TaSRG* and *TaSC* Genes

**Figure 3:**
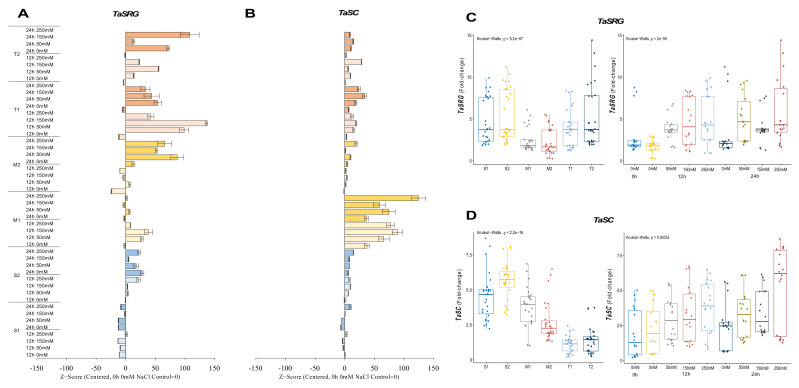
The expression of *TaSRG* and *TaSC* genes. (**A**) The gene expression pattern of *TaSRG* and (**B**) *TaSC* genes; (**C**) the total gene expression profile of *TaSRG* for genotypes, salt stress, and time course; (**D**) the total gene expression profile of *TaSC* for genotypes, salt stress, and time course. The Cq values formed the basis of qRT-PCR. The reference gene *TaACTIN* was used to standardize the Cq value for each sample. Z-score centered to 0 h 0 mM treatment (Control = 0). Values represent the means ± SD (*n* = 3).

**Figure A3:**
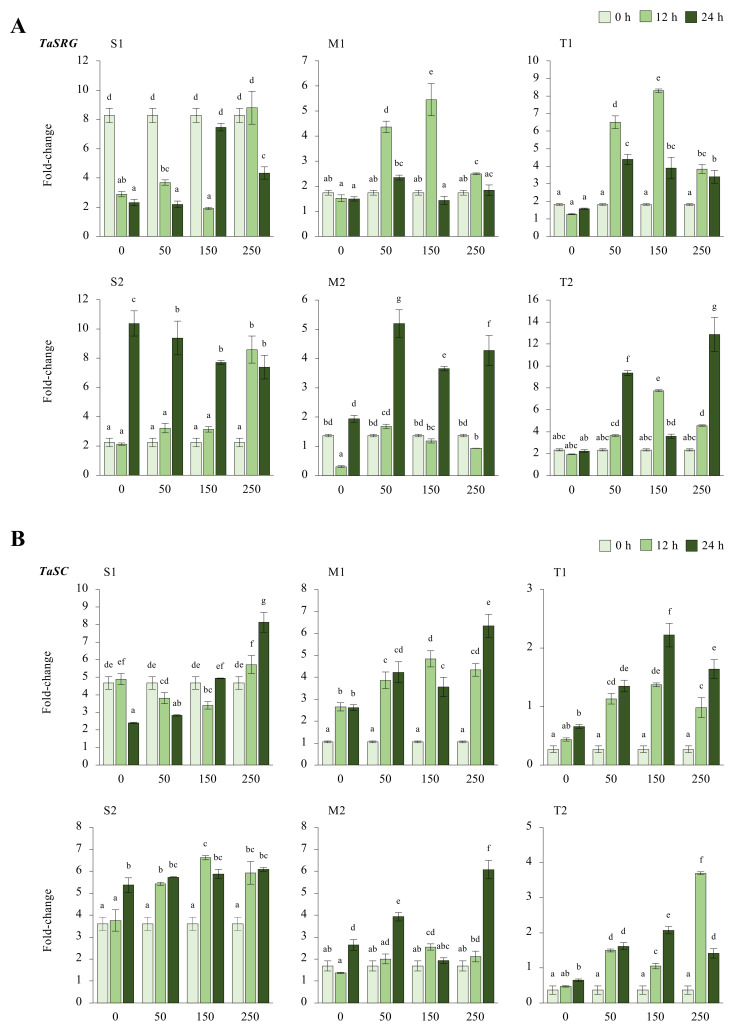
(**A**) The gene expression of the *TaSRG*, and (**B**) *TaSC* in the leaves of salt-sensitive (S1), S2, -moderate (M1), M2, -tolerant (T1), and T2 genotypes exposed to 0, 50, 150, and 250 mM NaCl stress for 0, 12, 14 h. The Cq values formed the basis of qRT-PCR. The reference gene *TaACTIN* was used to standardize the Cq value for each sample. Means (±standard deviation) within the same graph followed by letters are significantly different at *p* < 0.05 according to the Tukey HSD test from independent biological triplicates (*n* = 3).

The *TaSRG* transcripts were decreased from 8.2-fold at control up to 1.9-fold under salt-stress conditions, except for 12 h of 250 mM, which was slightly increased from 8.2-fold (control) to 8.7-fold in the S1 genotype. Although a high fold change in the mRNA transcript level was recorded at 24 h under control and salt-stress conditions in the S2 genotype, under salt-stress conditions, it was decreased from 10.3-fold (control) to 9.3-, 7.7-, and 7.3-fold under 50, 150, and 250 mM NaCl stress conditions, respectively. In addition, only a significant increment (ca. 6.3-fold and up) was recorded in 12 h of 250 mM NaCl stress. The M1 genotype showed the highest increment from 1.7-fold (control) to 4.3- and 5.4-fold in the *TaSRG* gene transcript at 12 h of 50 and 150 mM NaCl stress conditions, respectively. The expression of *TaSRG* was increased from 1.3-fold (control) to 5.1- and 4.2-fold at 24 h of 50 and 250 mM NaCl stress in the M2 genotype, respectively. The expression of *TaSRG* was increased in all salt-stress applications and time courses in the T1 and T2 genotypes. The highest increments were recorded as 8.2-fold at 12 h of 150 mM NaCl stress in the T1 genotype and 12.8-fold at 24 h of 250 mM NaCl stress in the T2 genotype (Figure 3A and Figure A3A). However, the T2 genotype displayed an even greater *TaSRG* expression profile. The overall *TaSRG* gene expression fold-change was shown to be lower in moderate genotypes and higher in sensitive genotypes. Under salt-stress conditions, the *TaSRG* transcript fold-change significantly increased, especially after 24 h of 250 mM NaCl stress (Figure 3C).

The transcript level of *TaSC* was decreased from 4.6-fold (control) to 2.8-fold under 50 mM NaCl for 24 h, but it was increased by 1.1- and 3.5-fold under 250 mM NaCl for 12 and 24 h, respectively, in the S1 genotype. The S2 genotype showed an increment in the transcript level of *TaSC* under all salt-stress conditions from 3.6-fold (control) up to 6.6-fold. The highest gene expression was observed to be a 6.6-fold increase at 12 h of 150 mM in the S2 genotype. Similarly, the gene expression of *TaSC* was increased in the M1 genotype under all salt-stress treatments and time courses. The highest gene expression was recorded as 4.3-fold and 6.3-fold under 250 mM NaCl stress at 12 and 24 h, respectively. The *TaSC* expression of the M2 genotype was increased in all stress applications, and the highest gene expression was found to be 6-fold in 24 h of 250 mM NaCl stress application. A similar pattern was observed in the T1 and T2 genotypes, in which *TaSC* gene expression was increased from 0.2-fold (control) to 2.2-fold (24 h of 250 mM NaCl) and 0.3-fold (control) to 3.6-fold (12 h of 150 mM NaCl) in the T1 and T2 genotypes, respectively (Figure 3B and Figure A3B). The total transcript level of the *TaSC* gene was found to be higher in the S2 genotype, and 12 and 24 h of 250 mM NaCl stress increased the gene expression fold-change of the *TaSC* gene (Figure 3D).

#### 2.2.3. *TaPIMP1* and *TaMIP* Genes

**Figure 4:**
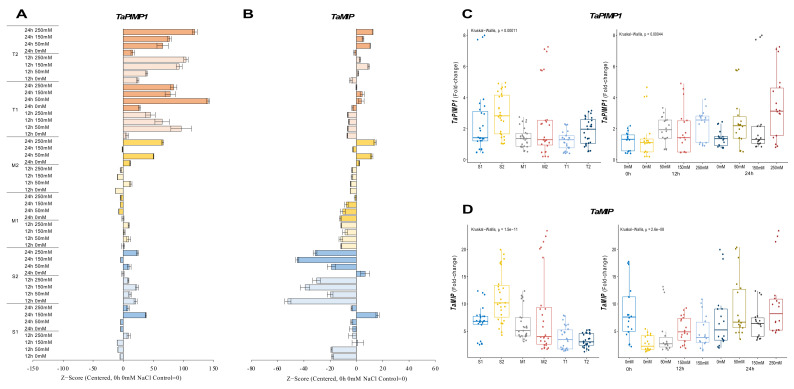
The expression of *TaPIMP1* and *TaMIP* genes. (**A**) The expression pattern of *TaPIMP1* and (**B**) *TaMIP* genes; (**C**) the total gene expression profile of *TaPIMP1* for genotypes, salt stress, and time course; (**D**) the total gene expression profile of *TaMIP* for genotypes, salt stress, and time course. The Cq values formed the basis of qRT-PCR. The reference gene *TaACTIN* was used to standardize the Cq value for each sample. Z-score centered to 0 h 0 mM treatment (Control = 0). Values represent the means ± SD (*n* = 3).

**Figure A4:**
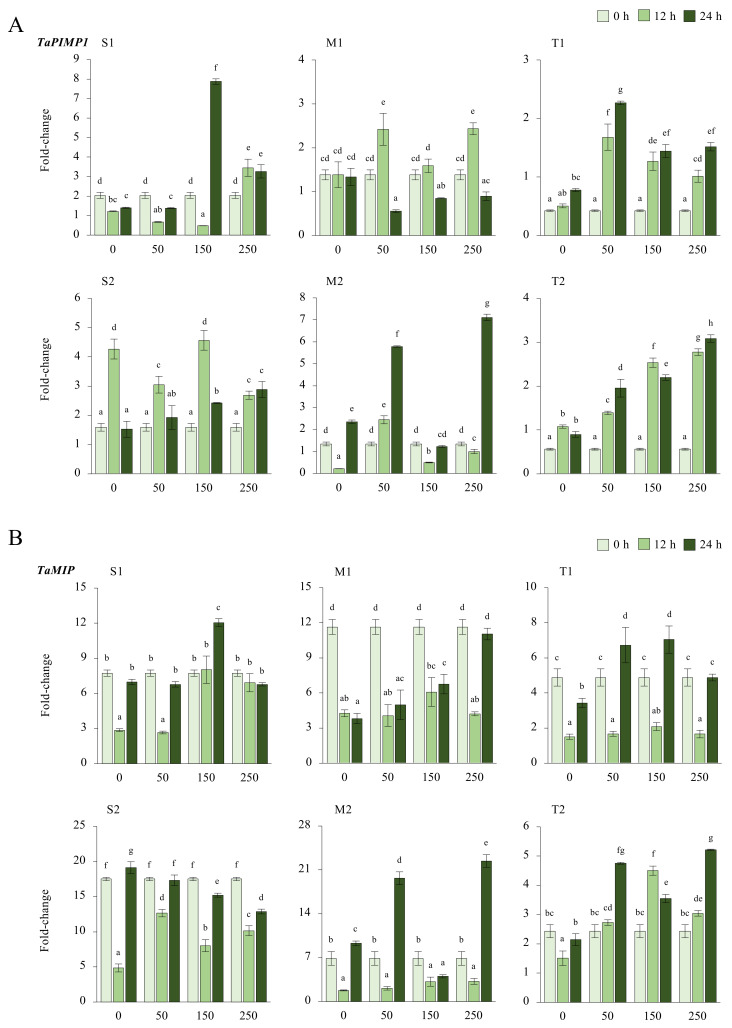
(**A**) The gene expression of the *TaPIMP1*, and (**B**) *TaMIP* in the leaves of salt-sensitive (S1), S2, -moderate (M1), M2, -tolerant (T1), and T2 genotypes exposed to 0, 50, 150, and 250 mM NaCl stress for 0, 12, 14 h. The Cq values formed the basis of qRT-PCR. The reference gene *TaACTIN* was used to standardize the Cq value for each sample. Means (±standard deviation) within the same graph followed by letters are significantly different at *p* < 0.05 according to the Tukey HSD test from independent biological triplicates (*n* = 3).

The expression of *TaPIMP1* significantly decreased in the S1 genotype under salt, except under 12 h of 250 mM NaCl and 24 h of 150 and 250 mM NaCl stress, resulting in 3.4-, 7.8-, and 3.2-fold increases, respectively. Although gene expression of *TaPIMP1* was increased in all salt treatments, it was decreased under 150 mM NaCl for 124 h. The *TaPIMP1* transcript level of the M1 genotype was found to be higher (2.4-fold) in 12 h of 50 and 250 mM NaCl stress conditions. The tolerant genotypes showed a significant increment in *TaPIMP1* expression under salt-stress conditions and time courses. The highest expression was recorded as 2.2-fold under 150 mM NaCl for 24 h in the T1 genotype and 3.6-fold at 12 h of 250 mM NaCl stress in the T2 genotype (Figure 4A and Figure A4A). The S2 genotype had overall fold-change increase in gene expression of *TaPIMP1*. Salt stress (250 mM NaCl) showed an increasing influence on the transcript fold-change of *TaPIMP1* gene after 12 and 24 hours of exposure. Higher salt doses and long-term exposure raised the *TaPIMP1* transcript fold-change in salt-sensitive and salt-tolerant genotypes (Figure 4C).

The transcript level of *TaMIP* was decreased in all salt applications and time courses in the S1 genotype, but only 12 h of 150 mM NaCl increased gene expression from 7.7-fold (control) to 12-fold. A similar pattern was observed in the S2 and M1 genotypes, where gene expression only increased from 17.2-fold (control) to 19.1-fold under 0 mM NaCl for 24 hours in the S2 genotype. The M2 genotype showed higher expression of 16.6-fold and 22.4-fold after 24 h of 50 and 250 mM NaCl stress application, respectively. *TaMIP* gene expression was decreased is the rest of the treatments compared to the control in the M2 genotype. The gene expression was increased from 4.8-fold (control) to 6.7- and 7-fold at 24 h of 50 and 250 mM NaCl in the T1 genotype, respectively. However, the T2 genotype showed a significant increment in *TaMIP* expression in almost all salt treatments and time courses, except 0 mM NaCl for 12 and 24 h, which are time control treatments (Figure 4B and Figure A4B). The total fold-change in gene expression was found to be higher in the S2 genotype. The 0 h of 0 mM and 24 h 250 mM NaCl treatments showed increments in the expression fold-change of *TaMIP* genes (Figure 4D).

#### 2.2.4. *TaHKT1;4* and *TaGSK1* Genes

**Figure 5:**
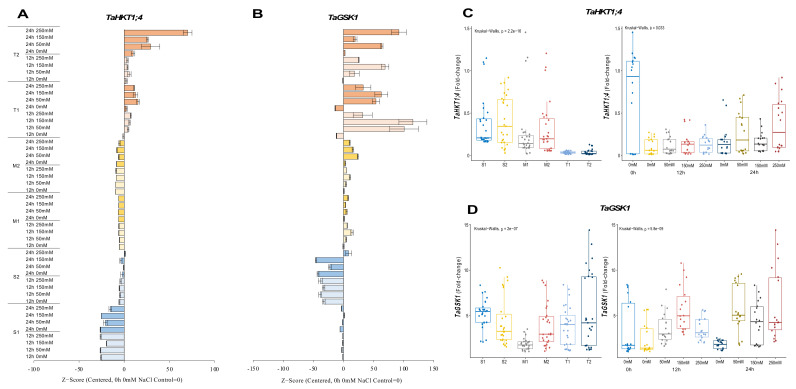
The expression of *TaHKT1;4* and *TaGSK1* genes. (**A**) The gene expression pattern of *TaHKT1;4* and (**B**) *TaGSK1* genes; (**C**) the total gene expression profile of *TaP5CS* for genotypes, salt stress, and time course; (**D**) the total gene expression profile of *TaGSK1* for genotypes, salt stress, and time course. The Cq values formed the basis of qRT-PCR. The reference gene *TaACTIN* was used to standardize the Cq value for each sample. Z-score centered to 0 h 0 mM treatment (Control = 0). Values represent the means ± SD (*n* = 3).

**Figure A5:**
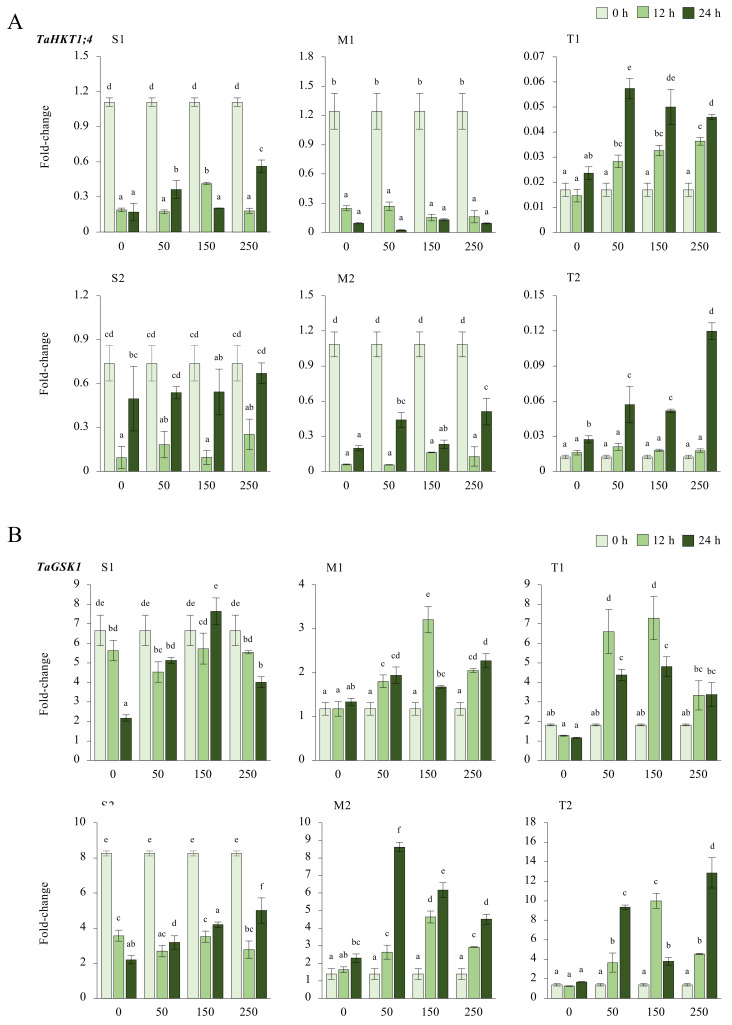
(**A**) The gene expression of the *TaHKT1;4* gene, and (**B**) *TaGSK1* in the leaves of salt-sensitive (S1), S2, -moderate (M1), M2, -tolerant (T1), and T2 genotypes exposed to 0, 50, 150, and 250 mM NaCl stress for 0, 12, 14 h. The Cq values formed the basis of qRT-PCR. The reference gene *TaACTIN* was used to standardize the Cq value for each sample. Means (±standard deviation) within the same graph followed by letters are significantly different at *p* < 0.05 according to the Tukey HSD test from independent biological triplicates (*n* = 3).

*TaHKT1;4* expression was significantly reduced for 12 and 24 h under all salt stress (50, 150, and 250 mM NaCl) applications in salt-sensitive and moderate genotypes. The expression was measured at a maximum of 0.5-fold under 250 mM NaCl for 24 h, but it was decreased by ca. 50% compared to 0 h of 0 mM NaCl conditions in the S1 genotype. The S2 genotype also showed a significant reduction in expression level from 0.7-fold (control) to 0.6-fold under 250 mM NaCl for 24 h. The transcript level of *TaHKT1;4* was decreased from 1.2-fold (control) to 0.2-fold (12 h of 50 mM NaCl) and from 1-fold (control) to 0.5-fold (24 h of 250 mM NaCl) in the M1 and M2 genotypes, respectively. The mRNA transcripts of *TaHKT1;4* were significantly increased in tolerant (T1 and T2) genotypes. The highest expressions were recorded as 0.057-fold and 0.050-fold at 24 h of 50 and 150 mM NaCl stress in the T1 genotype, respectively. Gene expression was increased at 24 h for all salt doses in T2 genotype. Higher *TaHKT1;4* expression was detected under 50 mM (0.057-fold) and 250 mM (0.12-fold) NaCl for 24 h in the T2 genotype (Figure 5A and Figure A5A). The total gene expression fold change of *TaHKT1;4* was found to be higher in the S2 genotype than in the other tested genotypes, and 0 h of 0 mM traits was the highest fold change in gene expression compared to other treatments **(**Figure 5C).

The expression of *TaGSK1* was decreased in the S1 genotype under all salt stress (50, 150, and 250 mM NaCl) treatments and time courses, but it was increased from 6.6-fold (control) to 7.6-fold under 24 h of 150 mM NaCl treatment in the S1 genotype. All salt treatments and time courses reduced the expression of *TaGSK1* in S2 genotype. The highest gene expression was recorded as 5-fold when the control (0 h of 0 mM NaCl) was 8.2-fold in the S2 genotype. The *TaGSK1* transcript level was increased in moderate (M1 and M2) genotypes under salt-stress conditions. The highest expression was observed as 3.2-fold under 150 mM NaCl stress for 12 h and 8.6-fold under 50 mM for 24 h in M1 and M2 genotypes, respectively. A similar pattern was observed in tolerant (T1 and T2) genotypes. The expression was increased from 1.8-fold (control) to 7.2-fold at 1 h of 150 mM NaCl stress in the T1 genotype, and it was increased from 1.3-fold (control) to 12.8-fold under 250 mM NaCl stress for 24 h in the T2 genotype (Figure 5B and Figure A5B). The total transcript level of *TaGSK1* was found to be higher in tolerant genotypes after 12 and 24 h (Figure 5D).

#### 2.2.5. *TaP5CS* and *TaMYB* Genes

**Figure 6:**
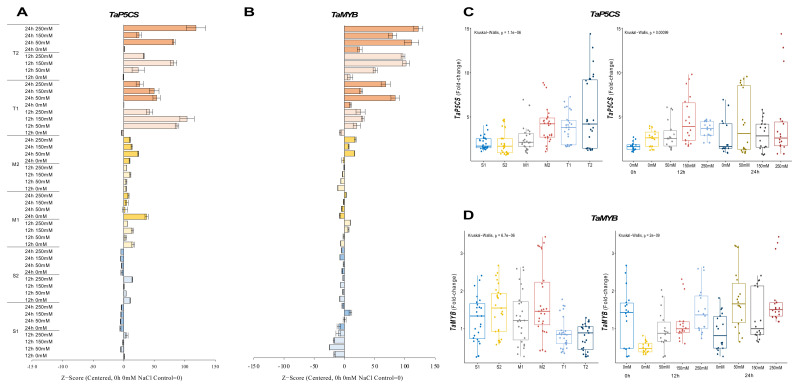
The expression of *TaP5CS* and *TaMYB* genes. (**A**) The expression pattern of *TaP5CS* and (**B**) *TaMYB* genes; (**C**) the total gene expression profile of *TaP5CS* for genotypes, salt stress, and time course; (**D**) the total gene expression profile of *TaMYB* for genotypes, salt stress, and time course. The Cq values formed the basis of qRT-PCR. The reference gene *TaACTIN* was used to standardize the Cq value for each sample. Z-score centered to 0 h 0 mM treatment (Control = 0). Values represent the means ± SD (*n* = 3).

**Figure A6:**
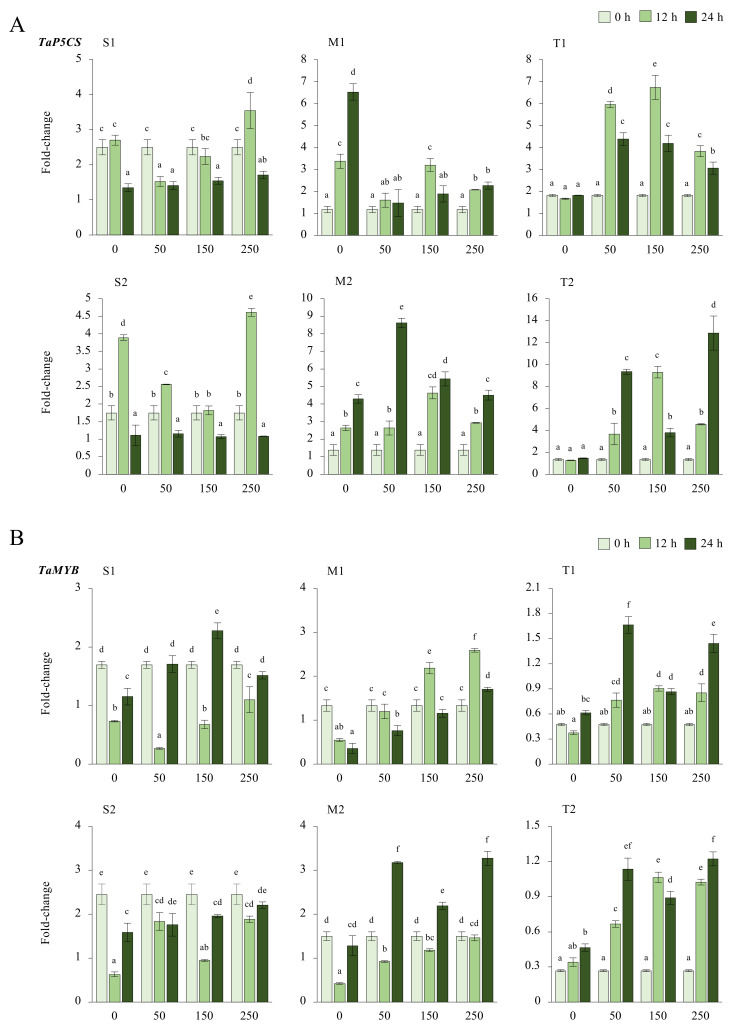
(**A**) The gene expression of the *TaP5CS*, and (**B**) *TaMYB* in the leaves of salt-sensitive (S1), S2, -moderate (M1), M2, -tolerant (T1), and T2 genotypes exposed to 0, 50, 150, and 250 mM NaCl stress for 0, 12, 14 h. The Cq values formed the basis of qRT-PCR. The reference gene *TaACTIN* was used to standardize the Cq value for each sample. Means (±standard deviation) within the same graph followed by letters are significantly different at *p* < 0.05 according to the Tukey HSD test from independent biological triplicates (*n* = 3).

The expression of *TaP5CS* was reduced in sensitive (S1 and S2) genotypes under salt-stress conditions, but it was increased from 2.4-fold (control) to 3.5-fold at 12 h of 250 mM NaCl stress in the S1 genotype and from 1.7-fold (control) to 4.6-fold under 250 mM for 12 h in the S2 genotype. The transcription of the *TaP5CS* gene was found to be more abundant in moderate (M1 and M2) genotypes under salt-stress applications. The transcript level of *TaP5CS* was increased from 1.7-fold (control) to 3.2-fold in the M1 genotype and from 1.3-fold (control) to 8.6-fold in the M2 genotype under 50 mM (12 h) and 150 mM (24 h) NaCl salt-stress conditions, respectively. A similar gene expression pattern was observed in tolerant (T1 and T2) genotypes. The highest expression was recorded as 6.7-fold and 12.8-fold in T1 (12 h of 150 mM NaCl) and T2 (24 h of 250 mM NaCl) genotypes (Figure 6A and Figure A6A). The total gene expression fold-change was found higher in the T2 genotype, and 12 and 24 h of salt exposure increased the expression fold-change of the *TaP5CS* gene under salt-stress conditions (Figure 6C).

The expression of *TaMYB* was significantly decreased in sensitive (S1 and S2) genotypes, with some exceptions. The expression was only increased from 2.4-fold (control) to 3.5-fold under 12 h of the 250 mM NaCl stress condition in the S1 genotype. *TaMYB* expression was found to be 2.1-fold and 2.5-fold higher at 12 h of 150 and 250 mM NaCl stress in the M1 genotype, respectively. The mRNA transcript level of *TaMYB* was found to be higher after 24 h compared to the 0 and 12 h periods. The highest gene expression was recorded as 3.2-fold at 24 h of 250 mM NaCl stress in the M2 genotype. The expression of *TaMYB* was increased by salt treatments (50 and 150 mM NaCl) in the tolerant (T1 and T2) genotypes. The highest gene expressions were observed as 1.6-fold and 1.3-fold at 24 h of 50 mM NaCl stress in the T1 and T2 genotypes, respectively (Figure 6B and Figure A6B). The total gene expression fold-change was found to be higher in the M2 genotype, but the effective activity of *TaMYB* was recorded as being higher after 24 h of salt-stress exposure (Figure 6D).

### 2.3. Principle Component, Hierarchical Clustering, and Correlation Network Analyses

**Figure 7:**
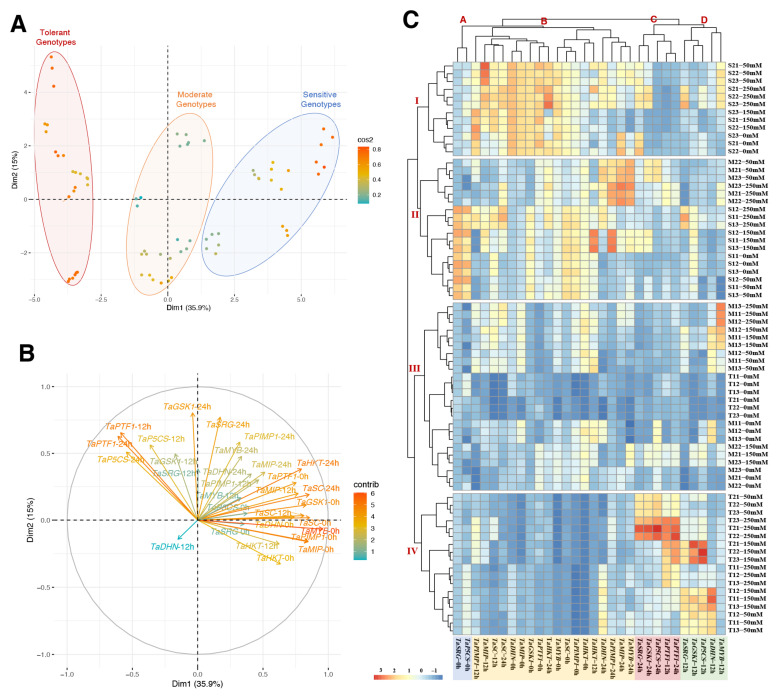
Principle component and hierarchical clustering analyses of genotypes and genes under salt stress and time course. (**A**) Principal component analysis (PCA) of the spatialization of two salt-sensitive, two moderate, and two tolerant genotypes (Colors blue: salt-sensitive, orange: moderate, and red: salt-tolerant genotypes), (**B**) PCA of the studied traits, (**C**) hierarchical clustering analysis (HCA) of measured gene expression level in salt-sensitive (S1, S2), moderate (M1, M2), and salt-tolerant (T1, T2) genotypes under 0, 12, and 24 h of 0, 50, 150, and 250 mM NaCl treatments. Clusters represent genotypes (I to IV), and genes (A to D).

The PCA analysis was performed on gene expression data from two salt-sensitive, two moderate, and two salt-tolerant wheat genotypes to evaluate the genes’ variables and identify the factors with a predominant influence on usability for the detection of salt-tolerant genotypes. The PCA exhibits those genotypes under all saline conditions, time courses, and variables associated with Dimmention1 (35.9%) and Dim2 (15%), of which Dim1 was the major component (total 50.9%) (Figure 7A and Table S3). The colors of the individual variables represent their quality of representation of the principal component abbreviated as ‘Cos2′. Almost all variables were expressed as high quality in the analysis. The tolerant, moderate, and sensitive genotypes were clearly separate from each other in Figure 7A. The salt-tolerant genotypes result in a high gene expression of the *TaPTF1* (12 and 24 h), *TaP5CS* (12 and 24 h), *TaGSK1* (12 and 24 h), and *TaSRG* (12 and 24 h) genes on the left side of the first axis (Dm1) (Figure 7B).

According to the heat map generated by the two-way hierarchical clustering analysis (HCA), all of the gene expression parameters under salt stress could be classified into four primary clusters (Figure 7C, groups A, B, C, D). A similar tendency has been observed for the genotypes after salt treatment. *TaP5CS* (0 h) and *TaSRG* (0 h) were separated into a distinct cluster (A) with high values in salt-sensitive genotypes. Cluster B showed the genes with higher values in sensitive and moderate genotypes under the control and salt-stress conditions. The *TaPTF1* (12 and 24 h), *TaP5CS* (12 and 24 h), *TaGSK1* (12 and 24 h), and *TaSRG* (12 and 24 h) genes were organized into two different clusters (C and D) with greater values under salinity conditions in tolerant genotypes, according to the HCA results. Under salinity stress, these characteristics are frequently reduced in salt-sensitive genotypes. The heatmap categorized the five germplasms into four separate clusters using data from salinity treatment (Figure 7C, groups I, II, III, IV). Under control and salt-stress conditions, Cluster I indicated the salt-sensitive genotype (S2). The M1 genotype under salt stress and the S1 genotype under control and salt-stress conditions were both assigned to cluster II. Cluster III represented the M1 genotype under both stress conditions, the M2 genotype under control conditions, and the tolerant genotypes under control conditions. Cluster IV was designated for only the salt-tolerant genotypes under salt-stress conditions.

**Figure 8:**
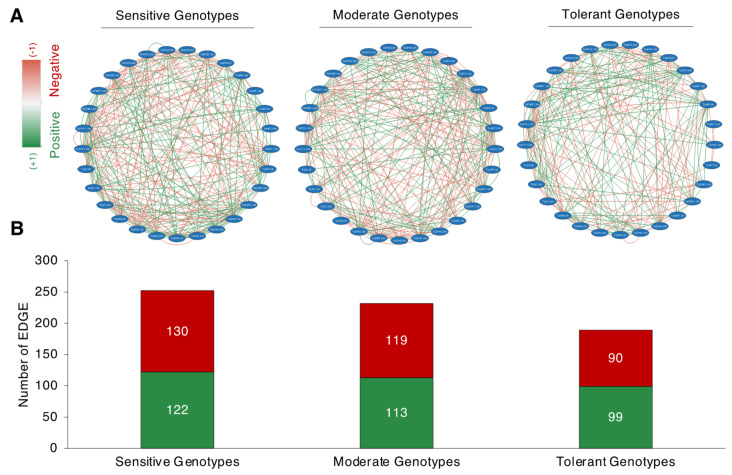
Correlation network analysis (CNA) of studied traits. (**A**) Correlation network analysis of analyzed traits of salt-sensitive (left panel), moderate (middle panel), and salt-tolerant (right panel) genotypes; (**B**) Positive and negative EDGE (total interaction) numbers of salt-sensitive, moderate, and salt-tolerant genotypes. Red (−1) and green (+1) colors correspond to the negative and positive edge associations, respectively (*p* < 0.05). An edge encodes an interaction or a tie between two traits.

Following the separation derived from the PCA, correlation-based network analysis (CNA) was constructed for sensitive, moderate, and tolerant genotypes, under normal salt-stress conditions (Figure 8A). The total interaction number (number of edges, where an edge encodes an interaction or a tie between two traits) was shown to be larger in the salt-sensitive and moderate genotypes but lower in the salt-tolerant genotypes. The salt-sensitive genotypes had 252 edges, with 122 positive correlations resulting in a positive edge-to-negative edge (pe/ne) ratio of 0.93. The moderate genotypes had 232 edges, with 113 corresponding to positive correlations and 119 corresponding to negative correlations, yielding a pe/ne ratio of 0.94. The salt-tolerant genotypes had a total of 189 edges, with 99 corresponding to positive and 90 corresponding to negative correlations, yielding a pe/ne ratio of 1.1. The CNA discovered a similar pattern across salt-sensitive and moderate genotypes, with both having a higher negative edge than a positive edge. The salt-tolerant genotype, on the other hand, has a higher positive edge than a negative edge.

## 3. Discussion

Rising sea levels and wheat farming irrigation systems based on underground water have increased soil salinity and reduced wheat productivity. Wheat is seriously affected by salinity concentrations in soils. Approximately 34% of wheat cropland is currently irrigated, 80–126 million ha of modern rainfed wheat cropland do not have enough discharge to meet irrigation demand, and 30 to 47% of today’s rainfed wheat croplands need more irrigation. On a global scale, more than half of all wheat cropland (62%) exhibits evapotranspiration, which is a typical case of deficit irrigation [42]. Saline groundwater up to 6 dSm^−1^, from which wheat can still draw 40% of its needs, has been shown in certain trials to diminish yield by 30% [43]. According to a different study, under the salinity thresholds of 4 and 15 dSm^−1^, there are, correspondingly, 10–55% decreases in wheat grain yield [11]. When combined with environmental circumstances, genetics is thought to be one of the most important aspects defining a plant species’ ability to tolerate salt [44]. As a result, the determination of salt-tolerant genotypes is a critical and time-consuming undertaking for increasing agricultural output. Although various promising morphological, physiological, and molecular marker strategies for determining stress-tolerant genotypes are under development, the molecular marker strategy remains one of the fastest detection methods at present.

Although numerous genes have been identified as molecular marker genes for salinity tolerance in the wheat genome, ion accumulation, and proline synthesis are the key molecular pathways for salinity tolerance in plants [45,46,47]. Different growth stages of plants exhibit variable degrees of salt stress, with the seedling stage in wheat being the most vulnerable because this stage determines the formation of the tillers, which, in turn, determines the number of spikelets and, eventually, the yield [48]. The response of plant growth to salinity often occurs in two stages: a quick response to a rise in osmotic pressure, followed by a more gradual response following the accumulation of Na^+^ in mature tissues, which results in ion toxicity that affects plant growth and development [2]. Numerous physiological changes are brought on by osmotic stress, such as cell membrane distortion, protein aggregation, DNA damage, disorganized ROS generation, severe ion imbalance, and reduced photosynthetic activity [49].

Under environmental stressors, proline serves as an effective osmolyte, antioxidative defense, and signaling molecule [50]. When plants are stressed, accumulating proline helps to maintain cell osmotic balance, stabilizes membranes to prevent electrolyte leakage, and acts as an antioxidant to control the level of reactive oxygen species (ROS) [51]. Proline is synthesized from glutamate by 1-pyrroline-5-carboxylate synthase (P5CS) in the glutamate pathway [26]. In our experiment, salt-tolerant and moderate genotypes showed a significant increment in the transcript level of *TaP5CS* gene and proline content under salt-stress conditions (Figure 1 and Figure 5A). The salt-sensitive genotypes also increased proline content under control conditions, but the increment level was significantly lower in salt-sensitive genotypes (Figure 1A,B). The time course did not significantly affect proline content in almost all genotypes, but salt doses significantly increased proline content in all genotypes. Hien et al. [52] showed a relative rise in proline concentration in saline-tolerant rice cultivars 48 h after stress application, while no increases were observed in the sensitive cultivar, even after 72 h of treatment with 200 mM NaCl. These genotype-specific responses may be related to signaling cascades that regulate proline metabolism, which is controlled by diverse cellular mechanisms and should be explored further. Among the factors involved, transcription factors, ion accumulation or balance, and abscisic acid (ABA) involved in gene signaling and expression related to proline biosynthesis [53].

Transcription factors are the key genes that initiate and regulate the transcripts of the genes. The MYB proteins are one of the largest transcription factor families in plants [34]. *TaMYB* is a key regulator gene involved in wheat salt-stress adaptation [35]. In our experiment, salt-tolerant genotypes showed increasing expression levels of *TaMYB* gene under salt-stress conditions (Figure 6B,D). The overexpression of *MYB2* genes increased proline accumulation in rice plants under salt-stress conditions [54]. Furthermore, a putative activation domain rich in acidic amino acid residues, glutamic acid, and aspartic acid significantly up-regulates *TaMYB* gene expression in drought and salt stress [35,55]. One other member of the R2R3-MYB transcription factor subfamily gene is *TaPIMP1*, which contains MYB DNA binding domains and enhances drought and salinity tolerance in plants [56]. The transcription level of the *TaPIMP1* gene in salt-tolerant genotypes was increased under salt-stress conditions compared with salt-sensitive and moderate genotypes (Figure 4A,C). *TaPIMP1* is controlled by ABA, and the overexpression of *TaPIMP1* increased proline synthesis, resulting in greater drought tolerance [36]. The overexpression of *TaPIMP1* improves salinity tolerance by enhancing chlorophyll content and superoxide dismutase (SOD) activity [56]. The most used genes in wheat are high-affinity potassium transporters (HKTs) that are responsible for Na^+^ and K^+^ ion homeostasis in wheat during salt stress [57]. The HKT family of proteins are expected to be important in plant salt-stress tolerance [58,59]. In our experiments, the expression level of *TaHKT1;4* genes were increased in salt-tolerant genotypes, and it was reduced in salt-sensitive genotypes under salt-stress conditions. However, the total expression level was found higher in salt-sensitive genotypes under salt-stress conditions (Figure 5B,D). *TaHKT1;4* has a higher functional variety among its members than the other HKT-type transporter groups and contributes to Na^+^ exclusion from leaf blades in response to salt stress. It was found to be expressed mostly in leaf sheaths and panicles [38,60,61].

Aquaporins (AQPs), which are water-selective channel proteins, mediate and control fast transmembrane water flow during activities such as seed germination, cell elongation, stomatal movement, phloem loading and unloading, reproductive growth, and stress responses [62]. According to protein subcellular location and amino acid sequence homology, the plant AQP family is split into three groups: small basic intrinsic protein (SIPs), nodulin 26-like intrinsic protein (NIPs), and plasma membrane intrinsic protein (PIPs) [63,64,65,66]. *TaMIP*, or *TaNIP*, is a novel aquaporin gene whose overexpression accumulates higher K^+^ and proline content and lower Na^+^ concentration in salt-exposed plants [19]. The salt-sensitive and moderate genotypes showed decreasing expression levels of *TaMIP*, but the expression level of *TaMIP* was increased in salt-tolerant genotypes under salt-stress conditions (Figure 4B,D). Additionally, the expression of several PIP-type genes was elevated in maize 2 h after 100 mM NaCl exposed maize for 2 h by collecting ABA [67]. Another study showed that a salt-tolerant rice genotype showed higher expression levels of aquaporin genes (*OsTIP*s and *OsPIP*s), *OsP5CS*, and proline accumulation under salt-stress conditions [68]. In response to salt, proline buildup is preceded by a rapid increase in the mRNA levels of *TaP5CS* and *TaDHN* genes, which regulate the rate-limiting stage of glutamate-derived proline biosynthesis [40]. The transcript level of the *TaDHN* gene was significantly increased in salt-tolerant genotypes under salt-stress conditions (Figure 3B,D), and the expression level of the *TaP5CS* gene was reduced in salt-sensitive genotypes but significantly increased in salt-tolerant genotypes under salt-stress conditions (Figure 6A,C). Transgenic plants demonstrate enhanced P5CS activity due to DHN-5 genes accumulating substantial amounts of proline [69].

*TaP5CS* transcript accumulation is tissue-specific and can be induced by salt and ABA. *TaSRG*, *TaPTF1*, *TaSC*, *TaPIMP1*, and *TaGSK1* genes were discovered to regulate osmotic stress via ABA signaling [20,21,36,70]. The expression level of the *TaSRG* gene was increased after 24 h of NaCl exposure in salt-tolerant genotypes (Figure 3A,C). *TaSRG* might also control P5CS gene expression, which could have helped the transgenic plants’ tolerance to salt by maintaining a high K^+^/Na^+^ ratio [20]. The basic helix–loop–helix transcription factors (bHLH) improve salinity tolerance in plants [37]. *TaPTF1* is a homolog of *OsPTF1*, a bHLH transcription factor, and *OsPTF1* has been demonstrated to provide tolerance to Pi deficiency in rice [71]. Previous studies showed that the transcript number of *TaPTF1* increased with the increase in *TaP5CS* and proline accumulation in salt-tolerant wheat genotypes [72]. In our experiment, the expression level of *TaPTF1* was increased in salt-tolerant and moderate genotypes, and it was decreased in salt-sensitive genotypes under salt-stress conditions (Figure 2A,C). *TaSC* overexpression in transgenic plants boosted free proline levels as well as P5CS gene expression. Under stress, ABA and NaCl increase *TaSC* gene expression [21]. The transcription number of *TaSC* was increased in salt-tolerant and moderate genotypes under salt-stress conditions, and the expression level of *TaSC* was found to be higher in salt-sensitive genotypes compared with salt-tolerant and moderate genotypes (Figure 3B,D). When salt stress was applied to the cells, the *TaSC* gene promoter perceived the ABA accumulation signal, which up-regulated its expression and increased the concentration of the second messenger Ca^2+^, finally activating the CDPK pathway. As a result of the expression of downstream genes in the pathway, proline accumulated, the intracellular K^+^/Na^+^ ratio increased, and chloroplast activity was improved [21]. A salt-inducible gene called *TaGSK1* exhibits a high degree of similarity with mammalian GSK3, a highly conserved serine/threonine protein kinase that controls the formation of glycogen [39]. In our experiment, the transcription level of *TaGSK1* was decreased in the salt-sensitive genotypes but significantly increased in salt-tolerant and moderate genotypes under salt-stress conditions (Figure 5B,D). Exogenously applied ABA and NaCl both stimulated *AtGSK1* expression, and *AtGSK1* overexpression improved NaCl tolerance in Arabidopsis [73]. Transgenic plants with overexpressed *TaGSK1* have reduced cellular osmotic turgor and Na^+^ concentration, as well as increased salt tolerance [39].

Previously, we found the wheat genotypes ‘Maycan’ (T1) and ‘Yildiz’ (T2) as salt-tolerant; ‘Kinaci-98’ (M1) and ‘Dogu-88’ (M2) as moderate; and ‘Esperia’ and ‘Sonmez-01’ as salt-sensitive [72]. Under salt conditions, the tolerant genotypes had higher osmoregulator proline content and antioxidant enzyme activity than the moderate and sensitive genotypes. All gene expression results demonstrated that salt-tolerant, salt-moderate, and salt-sensitive genotypes were categorized individually in the PCA analysis (Figure 7A), which is highly supported by our prior findings [72]. Under salt-stress conditions, salt stress raised the expression level of all studied genes in salt-tolerant genotypes. The salt-exposed time course revealed that 24 h of salt stress had a greater increasing effect on the gene expression levels of *TaPTF1*, *TaPIMP1*, *TaMIP*, *TaHKT1;4*, and *TaMYB* genes in salt-tolerant genotypes than 12 h did. Despite the fact that all genes related to proline accumulation and ion accumulation were studied, we found that the traits of *TaPTF1* (12 and 24 h), *TaP5CS* (12 and 24 h), *TaGSK1* (12 and 24 h), and *TaSRG* (12 and 24 h) were highly associated with tolerant genotypes (Figure 7C). Positive and negative correlations between the observed parameters indicate whether or not the investigated plant is stress-resistant. If negative correlations occur less often in the same plant than positive correlations, this suggests that the plant is salt-tolerant [74]. In our experiment, the correlation-network analysis revealed that salt-tolerant genotypes have a higher pe/ne ratio than salt-sensitive and salt-moderate genotypes (Figure 8B).

**Figure 9:**
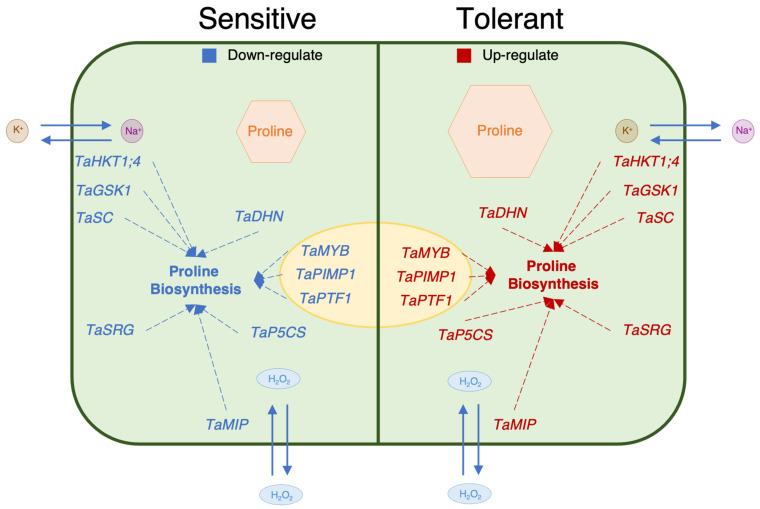
Suggested schematic illustration of a working model depicting the relationship between proline content and proline-related genes.

In the genotypes that are more tolerant to salt stress, the levels of transcript genes are more associated with the proline content [75]. The *TaSRG* may have influenced *TaP5CS* gene expression and increasing proline concentration in tolerant genotypes may be associated with increased *TaP5CS* gene transcript levels in tolerant genotypes as compared to sensitive genotypes [20,40]. The proline content of the genes encoding the transcription factors *TaMYB*, *TaPIMP1*, and *TaPTF1* increased during salt stress in salt-tolerant genotypes in the past [35,36,72]. In this investigation, we discovered that these transcription factors had higher levels of gene expression and enhance the proline concentration in genotypes that are tolerant. Another important mechanism for plants to tolerate salinity is the ion balance, which is controlled by the *TaHKT1;4*, *TaGSK1*, and *TaSC* genes. These genes also enhance proline concentration in plants under salt-stress conditions [21,39,70,76]. Aquaporin genes such as *TaMIP* play a crucial role in cell defense against salt stress with dehydration (*TaDHN*) genes, which is the major mechanism for salinity tolerance in plants after ion balance [18,67]. Additionally, prior research indicates that this gene contributes to proline accumulation in plants under salt stress [69]. Our findings demonstrated that, despite each gene’s unique role, improving proline biosynthesis served as a shared mechanism for differentiating between salt tolerance and sensitivity (Figure 9).

## 4. Materials and Methods

### 4.1. Plant Material and Experimental Design

Two salt-sensitive (S1; ‘Esperia’ and S2; ‘Sönmez-01’), moderate (M1; ‘Doğu-88’ and M2; ‘Kınacı-97’), and tolerant (T1; ‘Maycan’ and T2; ‘Yildiz’) wheat (*Triticum aestivum* L.) genotypes were used for the experiment [72]. The seeds were obtained from the Department of Breeding and Genetics, Central Research Institute for Field Crops, Ankara, Türkiye.

The experiment was performed with three independent replicates (each a pool of 5 plants). Seeds were surface-sterilized, rinsed three times with sterile distilled water, and germinated on moist blotting paper in plastic Petri dishes. After 3 days of incubation germinated seeds were transferred to 0.5 L pots filled with peat-based growing media (propagation substrate SF1, SuliFlor, Radviliškis, Lithuania) with the following characteristics: pH of 6, electrical conductivity (EC) of 0.65, 80% organic matter, and N-P2O5-K2O ratio of 14:16:18. Pots were placed in individual trays in a controlled growth chamber under light intensity equal to 27 μmol/m^2^/s. The temperature was maintained at 25/23 °C Day/night and relative humidity of 70%. Plants were irrigated with sterile water, and the same water level was maintained for all plants. Two-week-old wheat seedlings were subjected to 0, 50, 150, and 250 mM NaCl for 12 and 24 h.

### 4.2. RNA Extraction and cDNA Library

The total RNA was extracted from wheat leaves by using the TRizol method [77] according to the manufacturer’s instructions (Invitrogen, Waltham, MA, USA), following treatment with RNAase-free DNAase I (ThermoFisher, Waltham, MA, USA). Total RNA quantity and quality were determined using instructions from NanoDrop ND-1000 spectrophotometer (ThermoFisher, Waltham, MA, USA). The First Strand cDNA Synthesis Kit was used to generate cDNA templates from total RNA samples via reverse transcription (ThermoFisher, Waltham, MA, USA).

### 4.3. Real-Time Quantitative PCR

Transcript levels were analyzed in a CFX Connect™ 96 Real-Time PCR Detection System (Bio-Rad Laboratories GmbH, CA, USA). RT-PCR amplifications were conducted in a 15 µL reaction volume mixture containing 1.5 µL of cDNA, 4 µL ddH2O, 1 µL of 10 pmol forward (sense) primer, 1 µL of 10 pmol reverse (antisense) primer, and 7.5 µL iTaq™ Universal SYBER^®^ Green Supermix (Bio-Rad Laboratories GmbH, CA, USA). Each reaction for each gene was performed in triplicate following PCR protocol: 5 min 94 °C, 30 s 94 °C, 5 s 65 °C, 10 s 75 to 95 °C for melting curve (30 cycles). PCR amplification was performed with different cycles to ensure a linear response in the PCR. Primers used for RT-PCR are shown in Table 2, and their specificity was checked by separating the PCR products on 1.8% agarose gels. The *TaACTIN* (AB181991.1) gene was used as a reference gene, and the expression levels of genes were calculated by using the 2^−ΔΔCT^ method [78].

### 4.4. Proline Content

Free proline content was determined according to the method described by Bates et al. [79], with a slight modification. Fresh leaf samples were homogenized in 10 mL of 3% sulfosalicylic acid and incubated for 24 h at 4 °C. The homogenate was centrifuged at 10000 rpm at 25 °C for 5 min, and the supernatant (1 mL) was reacted with 1 mL ninhydrin reagent and 1 mL glacial acetic acid in a test tube at 100 °C for 1 h before stopping the reaction by immersing the tubes in an ice bath for 20 min. Proline was extracted with 2 mL of toluene and incubated for 30 min at room temperature. The toluene phase was separated, and absorbance at 520 nm was measured with a UVmini-1240 spectrophotometer (Shimadzu, Japan). Proline content was assessed from biological triplicated.

### 4.5. Evolutionary Analysis by Maximum Likelihood Method

The evolutionary history was interfered with by using the Maximum Likelihood method and Jones–Taylor–Thornton (JTT) matrix-based model [80]. The tree with the highest log likelihood (−2352.72) is shown. Initial tree(s) for the heuristic search were obtained automatically by applying Neighbor–Join and BioNJ algorithms to a matrix of pairwise distance estimated using the JTT model and then selecting the topology with superior log likelihood value. The tree is drawn to scale with branch lengths measured in the number of substitutions per site. This analysis involved 10 amino acid sequences. There was a total of 156 positions in the final dataset. Evolutionary analyses were conducted in the MEGA11.0.13 program [41,81].

### 4.6. Statistical, Principal Component, Hierarchical Clustering, and Network Analyses

The recorded data for each trait were initially standardized, obtaining the Z scores using means of the expression Z= (X − Y)/W, where Z is the value of the standardized variable corresponding to the respective trait and Y is the observation of 0 h and 0 mM NaCl. Y is the overall mean of the 0 mM 0 h trait in three replications, and W is the phenotypic standard deviation of the 0 mM 0 h trait. Collected data were subjected to a three-way analysis of variance (ANOVA) using R software (V4.2.1, https://www.r-project.org/, accessed on 28 November 2022) to assess differences among cultivars, salinity doses, and time course. Means separation was determined using Tukey’s honest significant difference (HSD) test at *p* < 0.05 with R software, including the ‘glht’ function in the ‘multcomp’ package [82]. Principal component analysis (PCA) was performed on the correlation matrix of 6 cultivars and transcript levels of genes under salt-stress conditions in a time course. Index values for each treatment were first calculated by assessing the salt-stress response vs. its control value. All the traits under each treatment were combined and used as index values for the PCA analysis. These index values were used to identify the correlation of response variable vectors and genotypes across the ordination space. A two-way heatmap clustering analysis (HCA) was performed on the same dataset as used in the PCA analysis. Pearson correlation was used as a correlation-based distance method, and ‘euclidean algorithm’ was used to compute the dissimilarity matrix. PCA and HCA were created using the R software, including the ‘prcomp’ function in the ‘factoextra’ package [83]. Data were hierarchically clustered using the heatmap function in the ‘pheatmap’ package with R software [84]. The correlation-based network analysis (CNA) was created according to Aycan et al. [74]. The CNA is displayed as a pairwise correlation comparison matrix. The genotypic correlation between traits (using salt-tolerant, moderate, and salt-sensitive groups) was estimated using Spearman’s correlation coefficient in R software. Topological properties of co-occurrence networks were analyzed using the Cytoscape software plugin NetworkAnalyzer (v3.9.0, Cytoscape Consortium, U.S.).

## 5. Conclusions

All genotypes had their proline content raised by salt stress; however, salt-tolerant genotypes had higher proline contents than the moderate and sensitive genotypes. Gene expression levels in salt-tolerant and moderate genotypes increased as a result of the salinity stress. *TaPTF1*, *TaPIMP1*, *TaMIP*, *TaHKT1;4*, and *TaMYB* genes were significantly upregulated after 24 h, whereas salt-stress exposure for 12 and 24 h had a major impact on gene expression in wheat. When compared to the salt-sensitive and salt-moderate genotypes, the salt-tolerant genotypes displayed a stronger positive than negative interaction. As a selective trait for salt-stress tolerance with proline synthesis, the *TaPTF1* (12 and 24 h), *TaP5CS* (12 and 24 h), *TaGSK1* (12 and 24 h), and *TaSRG* (12 and 24 h) genes can be employed. Our results showed that, despite each gene’s specific function, increasing proline biosynthesis functioned as a common mechanism for separating salt tolerance from sensitivity.

## Figures and Tables

**Table 1:** Analysis of variance (ANOVA) table.

Effect	T	S	T × S	T × G	S × G	T × S × G
Proline	***	***	***	***	***	***
*TaPTF1*	***	***	***	***	***	***
*TaDHN*	***	***	***	***	***	***
*TaSRG*	***	***	***	***	***	***
*TaSC*	***	***	***	***	***	***
*TaPIMP1*	***	***	***	***	***	***
*TaMIP*	***	***	***	***	***	***
*TaHKT1;4*	***	***	***	***	***	***
*TaGSK1*	***	***	***	***	***	***
*TaP5CS*	***	***	***	***	***	***
*TaMYB*	***	***	***	***	***	***

Time: T, Salt: S, Genotype: G, *** significant at *p* < 0.001.

**Table 2:** Primer sequence of genes used in the experiment.

Gene Name	Access. No.	Forward Primer (5’-3’)	Reverse Primer (5’-3’)
*TaPTF1*	DQ979392.1	GAAGCGAAAGGGAGTGGAATATTG	CCAAAAGATGAGATGCTACCACTG
*TaDHN*	FN393741.1	GTCCCGACTTCCCGTAGTTG	CCTTGATGTTCTCGCCGGTA
*TaSRG*	DQ672342.1	CGGAGATTGCACAGCGAAATTAAG	AAGCTTCTTCATCCTCATCCTCTC
*TaSC*	AY956330.1	CACACACGGACACCAAGTAATC	CCAGTATGTCAACCCGCTTATCAA
*TaMIP*	DQ530420	GTTCATCGATCCTCCTGACACAG	GAGATCATCGTCACCTTCAACATG
*TaPIMP1*	EU004200.1	TTCAGTCTCCTTATCTGGCATCTG	GCGACCAGAATGCCTAATATGTTC
*TaHKT1;4*	HG934161.1	AGCAAGCTGAAGTTGAGGGG	AGAGTTGTGACAGAGCCGTG
*TaGSK1*	AF525086.1	CATGGGTGGTTTGTTACATCGG	GACAATCTCAAACTCCTGGGGT
*TaP5CS*	KM523670.1	GAAGGCTCTTATGGGTGTACTCAA	TAAAAGACCTTCAACACCCACAGG
*TaMYB*	AY625680.1	GTAGGTGGTGAATGTGAAAGCTTC	GAGAATCGAAGCACAAGGGAAGTA
*TaACTIN*	AB181991.1	CAAAGAGATCACGGCCCTTG	CGGCATTGTCCACATGAAGT

## Data Availability

Not applicable.

## Supplementary Materials

The following supporting information can be downloaded at: https://www.mdpi.com/article/10.3390/plants11233401/s1, Table S1: Three-way analysis of variance (ANOVA) tables, *** significant at *p* < 0.001; Table S2: Amino acid sequences of *TaPTF1*, *TaDHN*, *TaSRG*, *TaSC*, *TaPIMP1*, *TaMIP*, *TaHKT1;4*, *TaGSK*, *TaP5CS*, and *TaMYB* genes; Table S3: Loading values and percentage contribution of variables on the axis identified by the principal component analysis (PCA) for all cultivars under control and saline conditions.
