# Supplementary material for: The TaGSK1, TaSRG, TaPTF1, and TaP5CS Gene Transcripts Confirm Salinity Tolerance by Increasing Proline Production in Wheat (Triticum aestivum L.)
Source: Plants (Basel). 2022 Dec 6;11(23):3401. doi: 10.3390/plants11233401 (PMC9738719; doi:10.3390/plants11233401)
Supplement: Supplementary file 1 [file plants-11-03401-s001.zip › Table S1.pdf]

Table S1. Three-way analysis of variance (ANOVA) tables, *** significant at $p < 0.001$.

TaPTF1

Effect	Dfn	DFd	F	*p*	*p* < 0.001	ges
**Time (T)**	2	144	1853.47	1.74E-103	***	0.963
**Salt (S)**	3	144	1256.525	5.15E-103	***	0.963
**Genotype (G)**	5	144	1574.685	9.41E-124	***	0.982
**T x S**	6	144	355.62	1.09E-83	***	0.937
**T x G**	10	144	731.449	4.43E-118	***	0.981
**S x G**	15	144	306.834	3.63E-101	***	0.97
**T x S x G**	30	144	88.714	3.07E-78	***	0.949

TaMIP

Effect	Dfn	DFd	F	*p*	*p* < 0.001	ges
**Time (T)**	2	144	979.914	1.40E-84	***	0.932
**Salt (S)**	3	144	82.288	4.59E-31	***	0.632
**Genotype (G)**	5	144	2027.243	1.59E-131	***	0.986
**T x S**	6	144	77.481	1.31E-42	***	0.764
**T x G**	10	144	341.972	5.04E-95	***	0.96
**S x G**	15	144	76.695	8.86E-61	***	0.889
**T x S x G**	30	144	80.909	1.52E-75	***	0.944

TaDHN

Effect	Dfn	DFd	F	*p*	*p* < 0.001	ges
**Time (T)**	2	144	85.899	2.78E-25	***	0.544
**Salt (S)**	3	144	144.151	3.54E-43	***	0.75
**Genotype (G)**	5	144	1414.29	1.85E-120	***	0.98
**T x S**	6	144	141.287	9.11E-58	***	0.855
**T x G**	10	144	659.719	6.37E-115	***	0.979
**S x G**	15	144	161.354	5.47E-82	***	0.944
**T x S x G**	30	144	69.684	3.30E-71	***	0.936

TaHKT1;4

Effect	Dfn	DFd	F	*p*	*p* < 0.001	ges
**Time (T)**	2	144	1219.965	5.22E-91	***	0.944
**Salt (S)**	3	144	10.562	2.54E-06	***	0.18
**Genotype (G)**	5	144	419.262	6.47E-84	***	0.936
**T x S**	6	144	10.169	2.29E-09	***	0.298
**T x G**	10	144	189.227	1.41E-77	***	0.929
**S x G**	15	144	3.358	7.47E-05	***	0.259
**T x S x G**	30	144	2.903	1.24E-05	***	0.377

TaSRG

Effect	Dfn	DFd	F	*p*	*p* < 0.001	ges
**Time (T)**	2	144	306.174	1.36E-52	***	0.81
**Salt (S)**	3	144	227.479	2.02E-54	***	0.826
**Genotype (G)**	5	144	460.853	1.09E-86	***	0.941
**T x S**	6	144	87.221	1.88E-45	***	0.784
**T x G**	10	144	308.845	5.57E-92	***	0.955
**S x G**	15	144	67.368	3.03E-57	***	0.875
**T x S x G**	30	144	79.162	6.60E-75	***	0.943

TaGSK1

Effect	Dfn	DFd	F	*p*	*p* < 0.001	ges
**Time (T)**	2	144	96.374	2.73E-27	***	0.572
**Salt (S)**	3	144	220.321	1.34E-53	***	0.821
**Genotype (G)**	5	144	383.828	2.42E-81	***	0.93
**T x S**	6	144	140.368	1.36E-57	***	0.854
**T x G**	10	144	240.059	1.61E-84	***	0.943
**S x G**	15	144	40.543	4.58E-44	***	0.809
**T x S x G**	30	144	44.955	8.44E-59	***	0.904

TaSC

Effect	Dfn	DFd	F	*p*	*p* < 0.001	ges
**Time (T)**	2	144	872.593	3.24E-81	***	0.924
**Salt (S)**	3	144	332.648	1.61E-64	***	0.874
**Genotype (G)**	5	144	1800.977	7.03E-128	***	0.984
**T x S**	6	144	114.005	3.72E-52	***	0.826
**T x G**	10	144	103.365	1.49E-60	***	0.878
**S x G**	15	144	24.025	6.51E-32	***	0.715
**T x S x G**	30	144	35.224	3.75E-52	***	0.88

TaP5CS

Effect	Dfn	DFd	F	*p*	*p* < 0.001	ges
**Time (T)**	2	144	780.821	5.08E-78	***	0.916
**Salt (S)**	3	144	72.612	1.16E-28	***	0.602
**Genotype (G)**	5	144	284.906	8.68E-73	***	0.908
**T x S**	6	144	92.669	6.15E-47	***	0.794
**T x G**	10	144	227.716	5.70E-83	***	0.941
**S x G**	15	144	111.39	3.01E-71	***	0.921
**T x S x G**	30	144	84.052	1.17E-76	***	0.946

TaPIMP1

Effect	Dfn	DFd	F	*p*	*p* < 0.001	ges
**Time (T)**	2	144	1050.396	1.31E-86	***	0.936
**Salt (S)**	3	144	321.544	1.35E-63	***	0.87
**Genotype (G)**	5	144	539.95	2.30E-91	***	0.949
**T x S**	6	144	128.495	2.92E-55	***	0.843
**T x G**	10	144	393.04	3.34E-99	***	0.965
**S x G**	15	144	147.978	1.87E-79	***	0.939
**T x S x G**	30	144	179.773	2.42E-99	***	0.974

TaMYB

Effect	Dfn	DFd	F	*p*	*p* < 0.001	ges
**Time (T)**	2	144	269.011	2.34E-49	***	0.789
**Salt (S)**	3	144	288.914	1.04E-60	***	0.858
**Genotype (G)**	5	144	590.295	5.16E-94	***	0.953
**T x S**	6	144	102.805	1.59E-49	***	0.811
**T x G**	10	144	262.056	4.18E-87	***	0.948
**S x G**	15	144	43.266	1.10E-45	***	0.818
**T x S x G**	30	144	25.232	1.90E-43	***	0.84

Proline

Effect	Dfn	DFd	F	*p*	*p* < 0.001	ges
**Time (T)**	2	144	1050.396	1.31E-86	***	0.936
**Salt (S)**	3	144	321.544	1.35E-63	***	0.87
**Genotype (G)**	5	144	539.95	2.30E-91	***	0.949
**T x S**	6	144	128.495	2.92E-55	***	0.843
**T x G**	10	144	393.04	3.34E-99	***	0.965
**S x G**	15	144	147.978	1.87E-79	***	0.939
**T x S x G**	30	144	179.773	2.42E-99	***	0.974
