# Supplementary material for: The TaGSK1, TaSRG, TaPTF1, and TaP5CS Gene Transcripts Confirm Salinity Tolerance by Increasing Proline Production in Wheat (Triticum aestivum L.)
Source: Plants (Basel). 2022 Dec 6;11(23):3401. doi: 10.3390/plants11233401 (PMC9738719; doi:10.3390/plants11233401)
Supplement: Supplementary file 1 [file plants-11-03401-s001.zip › Table S2.pdf]

Table S2. Amino acid sequences of TaPTF1, TaDHN, TaSRG, TaSC, TaPIMP1, TaMIP, TaHKT1;4, TaGSK, TaP5CS, and TaMYB genes

Gene name	Accession Number	Sequence
TaPTF1	ABI95371.1	MDYSNGSFFPSWPGNSASENYSFVDGSVESYAEEGSMPPTGYFRARSNQNLTFDEHEQNPAMLANGCLPY<br>NTQTDLLSGEILSEDKHSNSLMELPQLQNNGSLQSNLIPPGTLQCTSTPGTFDLQLDTPGLLELPHALSS<br>SIESNGSEVSAFLADVHAVSSASTLCSTFQNVPSYMEPVSLEAFSFQGIQNAAMFNNTSHSNGNLSVFDE<br>ATMASLHDSKEFLSGSISSFGTAEQSQLAGSGLKAEQQEQNAMCNIPLPFASGSQMAVSEAQGAMIPSKI<br>SSTMHNNKSEYPVPISHSADAQNKANSANGNSASAKPRARARRGQATDPHSIAERLRREKISERMKNLQD<br>LVPNSNKADKSSMLDEIIDYVKFLQLQVKVLCMSRLGAPGAVLPLLAESQTEGRSNSPLSSPTASQGLLD<br>AAGPEDSLVFEQEVIKLMETSITNAMQYLQNKGLCLMPIALASAISNQKGTSAAAIPPER
TaDHN	CAY85463.1	MEHQGHGAGEKKGVVESITEKLPGGHGDHQQATGGTYGQQGHAGVTGENIKEKLPGGHGDHQQTTGMTGS<br>ETHATTATTDGNYGKSGHTGTDGTGENKSIMDKIKDKLPGQH
TaSRG	ABG75754.1	MQGEVEQQTPMQMVLRVKHPSSLSSGSEEASEGEGSSRSALSVFKAKEEQIERKKMEVRDKVFAQLGRVE<br>EESKRLAFIRQELEGMADPTRKEVESIQRRIDTVNRQLKPLSKSCVKKEKEYKEILEAYNEKSKEKAILV<br>NRLIELVSESERMRMKKLEELNKTVDSLY
TaSC	AAY26392.1	MENLAMLWGIIGPGVAGALFGAGWWFWVDAVVCSAVQVSFIHYLPGIFASLAALMFNCVDKDAIGNDYYS<br>SYGDDSEWRVKLWLFVAYVVSFVCLAGSVGLLVQDALTDKGPSVWTGVAGVLQCVFVLISGLTYWTCHTE<br>D
TaMIP	ABF66147.1	MSVTSNTPTRANSRVNYSNEIHDLSTVQDGAPSLAPSMYYQEKSFADFFPPHLGKKVISEMVATFLLVFV<br>TCGAASIYGADVTRVSQLGQSVVGGLIVTVMIYATGHISGAHMNPAVTLSFACFRHFPWIQVPFYWAAQF<br>TGAMCAAFVLRAVLHPITVLGTTTPTGPHWHALVIEIIVTFNMMFITCAVATDSRAVGELAGLAVGSAVC<br>ITSIFAGPVSGGSMNPARTLAPAVASGVYTGLWIYFLGPVIGTLSGAWVYTYIRFEEEPSVKDGPQKLSS<br>FKLRRLQSQRSMAVDEFDHV
TaPIMP1	ABV59990.1	MTAGLQLGVIGSLALSVASSVAIVICNKALISTLGFPFATTLTSWHLMVTYCTLHVAQRLHFFEPKAIDG<br>HTVILFGFLNGTSIGLLNLSLGFNSIGFYQMTKLAIIPFTVLLETIFLNKRFSETIKLSLMVLLLGVGIA<br>SVTDLELNLLGSVLSGLAIATTCVGQILTNTIQKKLKVSSTQLLYQSAPYQAAILFATGPFVDRLLTNRS<br>VFAHKYTTPVVGFIILSCLIAVSVNFSTFLVIGTTSPVTYQVLGHLKTCLVLSFGYTLLHDPFTMKNILG<br>ILVAIFGMALYSFFSVRESKKKSTNDALPVSQMPDKETEPLLATKDGSDIKKANGVSHGC
TaHKT1;4	CDN24623.1	YLPPYTTWFPFEESSGVKDHPTEETQGSRLLKSTALSQLSYLAIFIIAICVTERENLEENPLNFSLLSIV<br>VEVVRQAPSNANQESLKTNNKYDCNLVPALLVSFMEADIF
TaGSK1	AAM77397.1	MGNMSIRDDRDPEDIVVNGNGTEPGHIIVTSIEGRNGQAKQTISYMAERVVGNGSFGTVFQAKCLETGET<br>VAIKKVLQDKRYKNRELQTMRVLDHPNVVALKHCFFSKTEKEELYLNLVLEYVPETAHRVIKHYNKMNQR<br>MPLIYAKLYMYQICRSLAYIHNSIGVCHRDIKPQNLLVNPHTHQLKLCDFGSAKVLVKGEPNISYICSRY<br>YRAPELIFGATEYTTAIDVWSAGCVLAELLLGQPIFPGDSGVDQLVEIIKVLGTPTREEIKCMNPNYTEF<br>KFPQIKAHPWHKIFHKRMPAEAVDLVSRLLQYSPSLRSTALEALIHPFFDELRDPNTRLPNGRFLPPLFN<br>FKPHELKGVPMDILVKLIPEHARKNCAFVGW
TaP5CS	AIZ77473.1	MAGADLNRSFIKDVKRIIIKVGTAVITRNDGRLALGRIGALCEQVKDLNAQGYEVIMVTSGAVGVGRQRL<br>RYRKLVNSSFADLQKPQMELDGKACAAVGQSGLMALYDMLFTQLDVSSSQLLVTDSDFDNSNFRERLRET<br>VESLLELRVIPIFNENDAISTRKAPYEDSSGIFWDNDSLAGLLALELKADLLVLLSDVDGLYSGPPSEPS<br>SKLIHTYIKEKHYHEITFGDKSRVGRGGMTAKVQAAVWASTGGVPVVITSGCASQSLVKVLRGEKIGTLF<br>HKNASLWEPSKDTSVREMAVAARDCSRRLQNLTSEERKKILVDVADALEANEDLIRSENEADLAAAHEAG<br>YESALVSRLTLKPGKIASLAKSVRTLANMEDPINEILKRTEVADGLVLEKTSCPLGVLLIIFESRPDALV<br>QIASLAIRSGNGLLLKGGKEAMRSNAILHKVITNAIPNNVGEKLIGLITTRDEIADLLKHDDVIDLVIPR<br>GSNKLVAQIKSSTKIPVLGHADGVCHVYIDKSADMDMAKRIVMDAKIDYPAACNAMETLLVHKDLMKTPE<br>LNDILVALKTAGVNLYCGPVAHKVLGYPKADSLHLEYSSMACTVEIVDDVQSAIDHIHRYGSAHTDCVVT<br>TDDKVAETFLRQVDSAAVLYNASTRFSDGARFGLGAEVGISTGRIHARGPVGVEGLLTTRWLLRGKGQVV<br>NGDKDVEYTHKSLPLQ
TaMYB	AAU06822.1	MMYHAKKFSVPFAPQRAQNSEHVSNIGAFGGSNISNPANPVGSGKQRLRWTSDLHSRFVDAIAQLGGPDR<br>ATPKGVLTVMGVPGITIYHVKSHLQKYRLAKYIPESPAEGSKDEKKDSSDSFSNADSAPGSQINEALKMQ<br>MEVQKRLHEQLEVQKQLQLRIEAQGKYLQMIIEEQQKLGGSLEGSEERKLSHSPPTLDDYPDSIQPSPKK<br>PRLDDLSTDAVRGVTQPGFESHLIGPWDQELCPKTNICDPAFQVDEFKANPGLSKS
