# Supplementary material for: The TaGSK1, TaSRG, TaPTF1, and TaP5CS Gene Transcripts Confirm Salinity Tolerance by Increasing Proline Production in Wheat (Triticum aestivum L.)
Source: Plants (Basel). 2022 Dec 6;11(23):3401. doi: 10.3390/plants11233401 (PMC9738719; doi:10.3390/plants11233401)
Supplement: Supplementary file 1 [file plants-11-03401-s001.zip › Table S3.pdf]

Table S3. Loading values and percentage contribution of variables on the axis identified by the principal component analysis (PCA) for all cultivars under control and saline conditions.

Variable	Dim. 1			Dim. 2			Dim. 3		
	Loading values	Contribution of variable (%)	Quality of representation	Loading values	Contribution of variable (%)	Quality of representation	Loading values	Contribution of variable (%)	Quality of representation
TaPTF1 0 h	5.271	0.754	0.568	1.742	0.280	0.078	0.051	-0.041	0.002
TaPTF1 12 h	3.234	-0.590	0.348	9.522	0.655	0.429	0.475	0.125	0.016
TaPTF1 24 h	3.458	-0.610	0.373	8.748	0.628	0.394	0.002	-0.007	0.000
TaDHN 0 h	5.060	0.738	0.545	0.012	0.023	0.001	7.475	-0.497	0.247
TaDHN 12 h	0.212	-0.151	0.023	0.453	-0.143	0.020	9.457	-0.559	0.312
TaDHN 24 h	1.559	0.410	0.168	2.664	0.346	0.120	1.246	-0.203	0.041
TaSRG 0 h	1.173	0.356	0.126	0.020	-0.030	0.001	14.654	0.695	0.484
TaSRG 12 h	0.000	0.002	0.000	3.266	0.384	0.147	4.616	-0.390	0.152
TaSRG 24 h	0.275	0.172	0.030	13.202	0.771	0.595	0.019	-0.025	0.001
TaSC 0 h	6.814	0.857	0.734	0.007	0.018	0.000	4.861	0.400	0.160
TaSC 12 h	6.151	0.814	0.663	0.025	0.034	0.001	0.892	-0.172	0.029
TaSC 24 h	6.770	0.854	0.729	0.812	0.191	0.037	0.914	-0.174	0.030
TaPIMP1 0 h	6.638	0.846	0.715	0.549	-0.157	0.025	3.711	0.350	0.122
TaPIMP1 12 h	2.025	0.467	0.218	2.015	0.301	0.091	8.752	-0.537	0.289
TaPIMP1 24 h	0.968	0.323	0.104	7.575	0.584	0.341	5.496	0.426	0.181
TaMIP 0 h	6.620	0.845	0.713	0.578	-0.161	0.026	5.844	-0.439	0.193
TaMIP 12 h	5.493	0.769	0.592	0.882	0.199	0.040	2.243	-0.272	0.074
TaMIP 24 h	2.452	0.514	0.264	2.857	0.359	0.129	0.312	0.102	0.010
TaHKT 0 h	3.725	0.634	0.401	2.371	-0.327	0.107	1.426	0.217	0.047
TaHKT 12 h	3.613	0.624	0.389	0.701	-0.178	0.032	1.802	0.244	0.059
TaHKT 24 h	5.869	0.795	0.632	3.258	0.383	0.147	0.029	-0.031	0.001
TaGSK1 0 h	6.420	0.832	0.692	0.285	0.113	0.013	0.259	0.093	0.009
TaGSK1 12 h	0.276	-0.173	0.030	5.469	0.496	0.246	0.352	0.108	0.012
TaGSK1 24 h	0.014	-0.039	0.002	14.336	0.804	0.646	1.301	0.207	0.043
TaP5CS 0 h	1.331	0.379	0.143	0.056	0.050	0.003	8.883	0.541	0.293
TaP5CS 12 h	1.231	-0.364	0.133	7.046	0.563	0.317	3.149	-0.322	0.104
TaP5CS 24 h	2.767	-0.546	0.298	5.765	0.510	0.260	0.183	0.078	0.006
TaMYB 0 h	8.513	0.958	0.917	0.101	-0.067	0.005	0.029	-0.031	0.001
TaMYB 12 h	1.019	0.331	0.110	0.623	0.168	0.028	7.877	-0.510	0.260
TaMYB 24 h	1.049	0.336	0.113	5.061	0.477	0.228	3.690	0.349	0.122
Eigenvalue		10.773			4.405			3.300	
Variability (%)		35.911			15.012			10.999	
Cumulative (%)		35.911			50.924			61.923	
